# Recent Developments of Cathode Materials for Thermal Batteries

**DOI:** 10.3389/fchem.2022.832972

**Published:** 2022-02-14

**Authors:** Renyi Li, Wei Guo, Yumin Qian

**Affiliations:** Key Lab of Advanced Optoelectronic Quantum Architecture and Measurement (MOE), School of Physics, Beijing Institute of Technology, Beijing, China

**Keywords:** thermal batteries, cathode materials, metal chlorides, metal fluorides, metal sulfides

## Abstract

Big progress has been made in batteries based on an intercalation mechanism in the last 20 years, but limited capacity in batteries hinders their further increase in energy density. The demand for more energy intensity makes research communities turn to conversion-type batteries. Thermal batteries are a special kind of conversion-type battery, which are thermally activated primary batteries composed mainly of cathode, anode, separator (electrolyte), and heating mass. Such kinds of battery employ an internal pyrotechnic source to make the battery stack reach its operating temperature. Thermal batteries have a long history of research and usage in military fields because of their high specific capacity, high specific energy, high thermal stability, long shelf life, and fast activation. These experiences and knowledge are of vital importance for the development of conversion-type batteries. This review provides a comprehensive account of recent studies on cathode materials. The paper covers the preparation, characterization of various cathode materials, and the performance test of thermal batteries. These advances have significant implications for the development of high-performance, low-cost, and mass production conversion-type batteries in the near future.

## Introduction

Thermal batteries are a special kind of primary batteries that can be stored in an inactive state for a long time and then activated generally at 350–550°C when energy is required (Guidotti and Masset, 2006; Choi et al., 2015). It was first invented and developed in the 1940s to power German weapons (Guidotti and Masset, 2006). The activation time and power density greatly affect the response speed of thermal batteries. Reserved thermal batteries with large capacity, high power, high thermal stability, long shelf lives, and fast activation are an attractive high-temperature battery system, which can be used as an effective power source for the actuator of guided weapons and the propulsion device of underwater and space vehicles (Cho et al., 2020).

**FIGURE 1:**
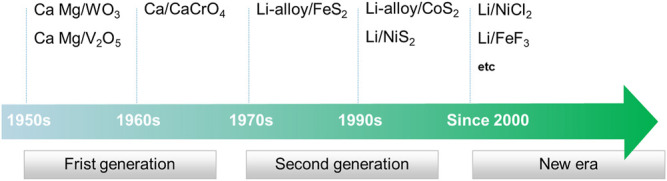
Historic development of thermal battery.

From the development history, thermal battery can be divided into three stages as shown in Figure 1. The first-generation calcium/chromate thermal battery had a low energy density, complex reaction mechanism, dendrite formation, and unpredictable performance, which is replaced by the second-generation lithium/sulfides batteries. Currently, the Li/FeS_2_ thermal battery with better power and discharging time compared with calcium/chromate counterpart, is widely used in various military weapons and environment. However, with the evolution of military action into an intelligence era and the thriving of space exploration, there are new challenges faced by batteries: energy/power density, discharging time, and activation time. Thus, the Li/FeS_2_ battery cannot meet the requirement and new battery technologies are essential to fulfill these needs. In terms of anodes, Ca, Mg, and Li or corresponding alloys all have been used as anode materials (Guidotti and Masset, 2008). However, only Li or Li-alloy is used at present, so this review only focuses on cathode materials related to Li chemistry. Higher voltage is more practical for a battery with a high-power output. Furthermore, obtaining the same voltage requires fewer single cells with high-voltage, which is beneficial to improve the overall energy density of the battery. Due to the stronger electronegativity of halide elements, halide compounds usually show higher voltages. Thus, metal halide materials are ideal replacement of metal sulfides as cathode materials for thermal batteries. This review highlights the reported metal sulfides and halides, and finally gives a generalization for the future research direction for cathode of thermal batteries.

## Transition Metal Sulfides

### FeS_2_

Pyrite is a relatively abundant and cheap natural mineral used as cathode material for thermal batteries (Guidotti et al., 2002). FeS_2_ has a capacity of 893 mAh/g with an open circuit voltage (OCV) of 2.0 V (Ko et al., 2019). It is a typical semiconductor with a bandgap of 0.95 eV (Ennaoui et al., 1993) and begins to decompose at 550°C. The synthesis of high-purity pyrite powder from natural mineral for thermal battery requires a complex ground, sizing, and purification process with high cost. The detailed preparation, characterization, and performance tests of FeS_2_ cathode are shown in Supplementary Figures S1–S5.

Guidotti used the plasma spraying (PS) method (Guidotti et al., 2006) to synthesize FeS_2_ with LiCl-KCl eutectic electrolyte to make the composite cathode, which showed low impedance and was almost 3.5 times (Guidotti et al., 2006) that of the discrete pressed-powder. The performance of batteries with PS cathode had a linear relationship with the number of cells, and the reduced battery components facilitate the battery design by reduced chance of assembly errors. Wang et al. synthesized pyrite films to solve mechanical integrity issues of thin pellets, and the prepared pyrite thin films with several superior physical properties than that of micron pyrite pellets (Au, 2003; Wang et al., 2013). Ko prepared thin-film cathodes with good homogeneity and a reproducible thickness *via* a tape-casting process (Ko et al., 2017), which can produce a thin sheet with a thickness of 0.01 to 1.2 mm, as well as the possibility of controlling density, surface condition, and the flexibility of synthesis sheets (Park et al., 2016). Such method can be easily scaled up, but increases the internal resistance of the battery due to the presence of different polymer binders (Masset et al., 2005; Cha et al., 2018). Various studies shows the phase transformations of FeS_2_ discharging process as follows (Tomczuk et al., 1982; Choi et al., 2014; Chen et al., 2017; Cha et al., 2018; Zhang and Tran, 2018; Kim et al., 2020; Zou et al., 2020; Thu et al., 2021): FeS_2_→Li_3_Fe_2_S_4_→Li_2+x_Fe_1-x_S_2_→Fe.

### CoS_2_

In special high-power and long discharging time applications, FeS_2_ is replaced by CoS_2_ due to its excellent thermal stability (650°C), metal-like electrical conductivity, and discharge performance (Preto et al., 1983; Guidotti et al., 2002; Guidotti and Masset, 2006). The capacity of CoS_2_ is 871 mAh/g, and OCV is 1.99 V^23^. The detailed preparation, characterization, and performance tests of CoS_2_ cathode are shown in Supplementary Figures S6–S8.

Guidotti prepared CoS_2_ cathode *via* an aqueous process (Guidotti et al., 2002) with improved thermal stability after heat treatment at 550°C. The performance can be enhanced by increasing the CoS_2_ particle size. Guidotti also showed that thermal spraying catholyte mixtures have distinct advantages for fabrication of thin electrodes for short-life thermal batteries (Guidotti et al., 2006). Xie prepared CoS_2_ and carbon nanotubes (CNTs) composite cathode using hydrothermal growth method (Xie et al., 2016). The pulse discharge performance of the CoS_2_/CNTs cathode was superior to that of conventional CoS_2_ due to the enhanced conductivity. Hu used the screening-printing method to prepare a film cathode (Hu et al., 2018) with a thickness of 50 μm that showed twice the capacity and active material utilization ratio. Xie prepared a novel carbon-coated CoS_2_ (C@CoS_2_) cathode material *via* a facile one-pot hydrothermal method (Xie et al., 2017) to prevent the CoS_2_ oxidation when exposing to air (Masset and Guidotti, 2008a; Masset and Guidotti, 2008b). The decomposition temperature of C@CoS_2_ was 610°C, which was 200°C higher than that of CoS_2_ nanocrystal (Masset and Guidotti, 2008a). Xie used carbon-coated CoS_2_ as the cathode to inhibit the higher self-discharge rate in the Li-B/CoS_2_ system (Xie et al., 2018).

There are many phases in the Co-S phase diagram, such as CoS_2_ (space group *Pa*-3), Co_3_S_4_ (space group *Fd*-3m), Co_9_S_8_ (*Fm*-*3m*), and CoS (*P*6_3_/*mmc*) (Masset and Guidotti, 2008b). Although the electrochemical performances of CoS_2_-based thermal batteries have been studied for many years, little is known about the phase evolution of CoS_2_ in the discharge process of thermal batteries. Previous studies showed that the phase transformation during the discharge process of CoS_2_ followed the CoS_2_→Co_3_S_4_→Co_9_S_8_→Co pathway (Masset, 2008). Payne’s powder neutron diffraction study showed that the phase transformations of the CoS_2_ discharging process followed the CoS_2_-CoS-Co_9_S_8_-Co pathway (Payne et al., 2019).

### NiS_2_

The low-cost NiS_2_ was regarded as one of the promising cathode material candidates because the thermal stability and discharge performance of NiS_2_ were between FeS_2_ and CoS_2_^27^. However, NiS_2_ cathode cannot fully react at high current density, which becomes a key problem for thermal batteries (Jin et al., 2018).

Nanocrystallization is an effective method to improve the specific capacity of cathode (Xu et al., 2014; Yu et al., 2016; Liu et al., 2017a; Yu et al., 2017). Moreover, the intermediate phase evolution and reaction speed could benefit from the merit of nanostructured cathode materials (Sen and Mitra, 2013). The more discharge steps appeared in the discharge curve, the more complete the NiS_2_ cathode reaction. Jin prepared nanostructured NiS_2_ using nickel carbonyl and sulfur powder as precursors by ball milling, which resulted in a double increase in specific capacity (Jin et al., 2017a).

Although nanocrystallizaiton can be used to improve the intermediate reaction rate and utilization rate of NiS_2_, it decreases the decomposition temperature (Ji et al., 2014; Yang et al., 2014; Xie et al., 2016), which makes it suitable only for thermal batteries with short life time (discharge temperature ≤500°C). Jin employed hierarchical carbon modification to enhance the thermal stability and conductivity of nanostructured NiS_2_ with significantly higher discharge performance than the pristine NiS_2_ with much higher resistance (Jin et al., 2018). Hierarchical carbon modification not only increased the initial decomposition temperature but also prevented the further decomposition of residual NiS_2_. The characterization and performance tests of the nanostructured NiS_2_ powder are shown in detail in Supplementary Figure S9.

The Ni-S phase diagram exhibits various Ni_X_S_Y_ (X ≤ 7, Y ≤ 6) polymorphism structures (Payne et al., 2017). However, despite years of studies, little is known about how the crystalline phases in batteries evolve at high temperatures (Payne et al., 2017).

The HRTEM measurement confirms that the phase transformations of the NiS_2_ discharging process followed the NiS_2_→NiS→Ni_7_S_6_→Ni_3_S_2_→Ni pathway (Jin et al., 2017a). Payne’s *in situ* powder neutron diffraction study (Payne et al., 2017) showed that the phase transformations of NiS_2_ the discharging process followed the NiS_2_→NiS→Ni_2_S_3_→Ni pathway.

In recent years, transition metal dichalcogenides (TMDs) have received considerable attention due to their novel layer structure (Zhang et al., 2018) with unique properties, which endow great potential in catalysis (Liang et al., 2020; Sarma et al., 2020; Xu et al., 2020; Zeng et al., 2020; Wang et al., 2021), energy storage, and conversion (Gao et al., 2016; Li et al., 2019). Various TMDs have been reported as thermal battery cathode material with high decomposition temperature and capacity, such as MoS_2_ (Zheng et al., 2018), WS_2_ (Guo et al., 2019a), NiMoSs (Yusong Choi, 2019), and ZrS_3_ (Giagloglou et al., 2016). These materials show various advantage over traditional FeS_2_, which deserve detailed study in the future.

## Transition Metal Chloride

It is urgent to develop new cathode materials for thermal batteries with high power and energy output capability, miniaturization, and micromation to adapt to the rapid development of weapon and space exploration system (Tian et al., 2021), which is difficult for these traditional cathode materials (Liu et al., 2017b). The detailed synthesis, characterization, and performance tests of NiCl_2_ are exhibited in Supplementary Figures S10–S12.

NiCl_2_ is considered an ideal substitute cathode material for FeS_2_ and matches the excellent performance of Li alloy anode due to its relative high potential (2.5–3 V vs Li), high discharge current, high specific energy, and low cost (Guidotti et al., 2006; Ying et al., 2016; Liu et al., 2017b). However, the high solubility of NiCl_2_ in molten salt electrolyte can cause short circuit. Due to the low conductivity and poor electrochemical activity of NiCl_2_, the activation time is longer, so a certain amount of conductive additives must be added to improve its conductivity (Jai Prakash, 2000; Jin et al., 2017b; Gui et al., 2020; Tian et al., 2021).

The thermal stability of NiCl_2_ was primarily affected by the crystal water and oxide impurities during low-temperature pre-dehydration. Thus, surface and sublimation treatment of hydrate NiCl_2_ are necessary to modify the microstructure and make large specific surface area to boost the electrochemical performance. Jin prepared pure NiCl_2_ by vacuum sintering after sublimation and synthesized the carbon-coated NiCl_2_ *via* a simple solid-state reaction (Jin et al., 2017b). The carbon-coated NiCl_2_ cathode showed drastic improvement in capacity, specific energy, and discharging time. The electrochemical mechanism was shown as follows:

$$2\mathrm{Li}+{\mathrm{NiCl}}_{2}\to 2\mathrm{LiCl}+\mathrm{Li}$$

The carbon coating enhanced the electronic conductivity and led to the reduced activation time. At the same time, the carbon coating increased the structure stability and reduced the solubility of NiCl_2_ by protecting the NiCl_2_ from directly contacting the electrolyte. However, it also resulted in a greater voltage delay during the initial discharge process. Hu filled sublimated NiCl_2_ into Ni foam instead of compact stainless steel screen printing (Hu et al., 2017). The discharge voltage of the NiCl_2_-based single cell with Ni foam substrate was 2.55 V. Compared with NiCl_2_ cathode with stainless steel substrate, the electrochemical test showed that the NiCl_2_/Ni foam cathode had more than twice the capacity, had almost one-third of the internal resistance, and had a power density of 10.866 kW/kg, which outweighed the sulfide batteries. Ni foam substrate can enhance the electronic conductivity, causing the fast electron transfer and reaction (Xie et al., 2017). Liu proposed a novel two-step variable temperature solid-state method to remove the crystal water from NiCl_2_ hexahydrate (Liu et al., 2017b). The study showed that the sintering temperatures influenced the morphology of materials, which induced the reduction in discharging time and specific capacity. The two-step variable-temperature preparation method was an effective method to enhance the thermal stability of NiCl_2_ cathode materials. The 600°C-treated cathode exhibited better specific capacities of 210.42 and 242.84 mAh/g at 0.5 and 2.0 A. Giagloglou prepared NiCl_2_ by solid-state reaction in sealed evacuated quartz tubes, which showed enhanced performance (Giagloglou et al., 2018) and whose specific capacity was achieved at 360 mAh/g under 500°C. By using a simple hydrogen etching technique, Gui successfully modified the Ni content and surface roughness (Gui et al., 2020). At different etching conditions, the prepared NiCl_2_ showed higher discharge voltage and significantly shorter activation time than those of the pure NiCl_2_ cathode. The discharge voltage and specific power of the prepared NiCl_2_ *via* hydrogen etching technique were 2.43 V and 7.59 kW/kg. The fabricated the Ni-NiCl_2_ composite cathode material *via* hydrogen reduction by Tian et al. (2021) showed higher voltage and much shorter activation time, and half of the internal resistance and the power density was up to 11.4 kW/kg.

Additionally, PbCl_2_ was reported as cathode material (Chen et al., 2021). Although PbCl_2_ had a relatively low theoretical specific capacity (193 mAh/g), due to the high utilization rate, it still may increase the energy density of the battery. Giagloglou synthesized KNiCl_3_, Li_2_MnCl_4_, and Li_6_VCl_8_ *via* solid-state reaction and used them in the thermal battery (Giagloglou et al., 2018). Compared with well-known metal disulfide, these transition metal chlorides provided greater specific power and exhibited higher voltage, which can be considered as promising alternative materials for Li thermal battery applications.

## Transition Metal Fluorides

Transition metal fluorides (TMFs) have attracted much attention because of their high voltage, specific energy, and excellent thermal stability (Yamakawa et al., 2009; Kim et al., 2010; Chun et al., 2016; Sheng Nan Guo and Wang, 2019; Chang et al., 2020). The ionic bond property of TMFs results in a high working potential (Li et al., 2012). The theoretical potential of the most attractive cathode material, CuF_2_, is up to 3.55 V with a specific capacity as high as 528 mAh/g and a specific energy of 1.87 kW h/kg (Yamakawa et al., 2009). The theoretical potential of NiF_2_ is 2.96 V, and the specific capacity is 554 mAh/g. Chang prepared pure NiF_2_ *via* a direct and simple two-step dehydration method from commercial NiF_2_.4H_2_O (Chang et al., 2020). The discharge mechanism was as follows:

$$\mathrm{Ni}F_{2}+2\mathrm{Li}\to \mathrm{2LiF+Ni}$$

Their electrochemical tests of NiF_2_ cathode showed that there was a voltage platform approximately 2.4 V at 0.1 A/cm^2^ under 520°C. The maximum voltage plateau can reach 2.5 V when the temperature is raised up to 580°C, and the corresponding specific power was 3.7 kW/kg, making it possible to apply in high specific power thermal batteries. The voltage plateaus dropped in succession when the current density is increased due to the enhanced concentration polarization. The discharge time decreased from 295 to 88 s when the current density is increased from 0.1 A/cm^2^ to 0.5 A/cm^2^, which induced a lower specific capacity. The specific power reached up to 16.2 kW/kg at a high current density of 0.5 A/cm^2^, which proved that NiF_2_ can be used as the cathode material with high specific power and energy. Compared to the cathode material NiCl_2_ with long activation time and severe infiltration, the NiF_2_ exhibited better electrochemical performance. The average resistance was 0.56 Ω, which was equivalent to the total polarization. Carbon coating was an effective means to further improve the thermal stability and electrochemical performance of NiF_2_. The detailed synthesis, characterization, and performance tests of NiF_2_ are shown in Supplementary Figures S13, S14.

FeF_3_ is another investigated cathode material (Hua et al., 2021). During the lithiation process, two characteristic discharge voltages of FeF_3_ were exhibited at about 3 V and below 2 V, and the corresponding capacities were 237 and 175 mAh/g, respectively. The electrochemical mechanism of FeF_3_ follows a three-electron-transfer reaction (Badway et al., 2003):

$$\mathrm{Fe}F_{3}+{\mathrm{Li+e}}^{‐}\to \mathrm{LiFe}F_{3}$$

$$\mathrm{LiFe}F_{3}+2{\mathrm{Li+2e}}^{‐}\to Fe^{0}+3\mathrm{LiF}$$

Guo synthesized the anhydrous FeF_3_ by a liquid-phase method combined with a thermal treatment process of the FeF_3_.3H_2_O/MWCNTs composite (Guo et al., 2019b). During heat treatment, the FeF_3_.3H_2_O crystal smooth surface became rough due to the release of crystal water and phase transformation, and more particles were broken into small nanoparticles. However, MWCNT fibers can still be evenly distributed among FeF_3_ particles by this simple and convenient method. The large bandgap of pristine FeF_3_ can hinder the transfer electrons, inducing more self-discharge reaction than electrode reaction. The electrochemical test showed that conductive MWCNTs can facilitate electron transfer, and the electrode reaction prioritized the self-discharge reaction. The additive MWCNTs led to a decrease of the total polarization of FeF_3_ from 45 to 10 mΩ, proving that the MWCNTs can form a conductive network among FeF_3_ particles and greatly improve the conductivity. Wang used a scalable and low-cost strategy to prepare a CoF_2_/CNTs cathode nanocomposite, and the capacity was achieved at 550 mAh/g with excellent mechanical properties (Wang et al., 2015). Other TMFs, such as FeF_2_ (Wang et al., 2011), TiF_3_ (Kitajou et al., 2017; Kitajou et al., 2019), and MnF_3_ (Read, 2012), have been successfully synthesized and exhibited excellent performance. The summary of the cathode type, size/thickness, phase, and performance of cathode materials in recent studies is shown in Supplementary Table S1.

## Summary and Outlook

**FIGURE 2:**
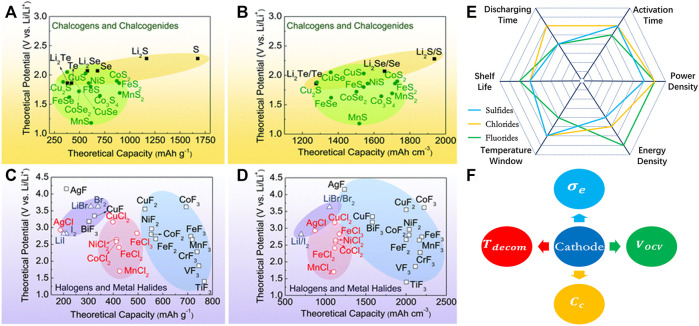
Theoretical gravimetric and volumetric capacities and theoretical potential of selected conversion cathode materials: **(A,B)** chalcogens and chalcogenides; **(C,D)** halogens and metal halides (reproduced from Wu and Yushin, 2017, Copyright 2017 Royal Society of Chemistry). **(E)** Comparison for the various properties of thermal batteries using sulfides, chlorides, and fluorides as the cathode material. **(F)** The future development directions for the high-performance cathode material of thermal battery.

Theoretic calculation is another tool for the design of high-performance cathode materials as shown in Figures 2A–D; many new materials have been designed to be potential candidates (Wu and Yushin, 2017). The strength and weakness of metal sulfides, chlorides, and fluorides are summarized and compared as shown in Figure 2E. Currently, sulfides are most studied and widely used as cathode materials for thermal batteries, but they do not meet the new challenge with the development of military weapons and space exploration in the future. Chlorides and fluorides have relatively high voltage and decomposition temperature, which make them suitable for the high-energy-density and long-discharging-time battery. However, they are still at the infancy stage; more detailed studies need to be carried out for the real application.

For the future development of cathode materials for thermal batteries, they can be improved mainly from the following four aspects, as shown in Figure 2F:

1) High voltage V_ocv_: this is the most important factor that influences the power and energy density of the thermal battery. More importantly, a higher-voltage cell will use fewer series cells to achieve the demanding output voltage and a simpler battery management system. Thus, the higher effective mass ratio of active material is also helpful for further enhancement of energy density. In this respect, the metal fluoride has overall advantage over chloride, sulfide, or oxides, because of the higher electronegativity difference between fluorine and alkali metal.
2) High capacity C_c_: transition metal compound with multiple valency metal and nonmetal element will have multiple electron transfer reaction with a relative higher n/M ratio, which leads to a higher capacity. Transition metals with high valency state compounds are potential materials
3) High decomposition temperature T_decom_: thermal battery discharging time depends on the lower and upper temperature limit of the working temperature window, T_dw_ and T_up_, which are usually determined by the melting point of electrolyte and the decomposition temperature of cathode, respectively. Usually, electrolyte is a mixture of halide salts with very high boiling temperature and decomposition temperature. So, the cathode decomposition temperature is usually T_up_. The larger the T_up_ − T_dw_ difference, the longer the discharging time. Nanostructure materials often show lower T_decom_, but surface modification has shown increased T_decom_.
4) High electron conductivity *σ*_e_: the cathode that has higher electron conductivity will facilitate the ion/electron transfer and thus has low internal resistance and higher output voltage. More importantly, it can yield larger current and thus has a large output power. However, almost all these metal compounds are insulators; thus, proper surface or doping engineering is necessary to improve the conductivity.

Coating, doping, and tailoring the microstructure are effective methods to improve the discharge performance of cathode (Tan et al., 2016). Carbon modification is an effective method to improve the stability and conductivity of electrode materials (Oh et al., 2010; Chinnappan et al., 2016; Jiang et al., 2016; Li et al., 2016; Shan et al., 2016; Zhang et al., 2016; Liu et al., 2017c; Li et al., 2017). In real application, simultaneously improving these factors without hindering other factors is impossible. Optimizing the cell design or electrode designs, such as tape-casting and screening-printing, to increase the mass ratio of active material is another useful way to upgrade the energy density of batteries (Ko et al., 2017).
