# Supplementary material for: Recent Developments of Cathode Materials for Thermal Batteries
Source: Front Chem. 2022 Feb 14;10:832972. doi: 10.3389/fchem.2022.832972 (PMC8882827; doi:10.3389/fchem.2022.832972)
Supplement: Supplementary file 1 [file DataSheet1.PDF]

Supplement Information

Recent Developments of Cathode Materials for Thermal Batteries

Renyi Li[1], Wei Guo[1*], Yumin Qian[1*]

[1]Key Lab of Advanced Optoelectronic Quantum Architecture and Measurement (MOE), School of Physics, Beijing Institute of Technology, Beijing 100081, China

[*] **Correspondence:**

Corresponding Author

E-mail:

weiguo7@bit.edu.cn

yuminqian@bit.edu.cn

1. FeS_2

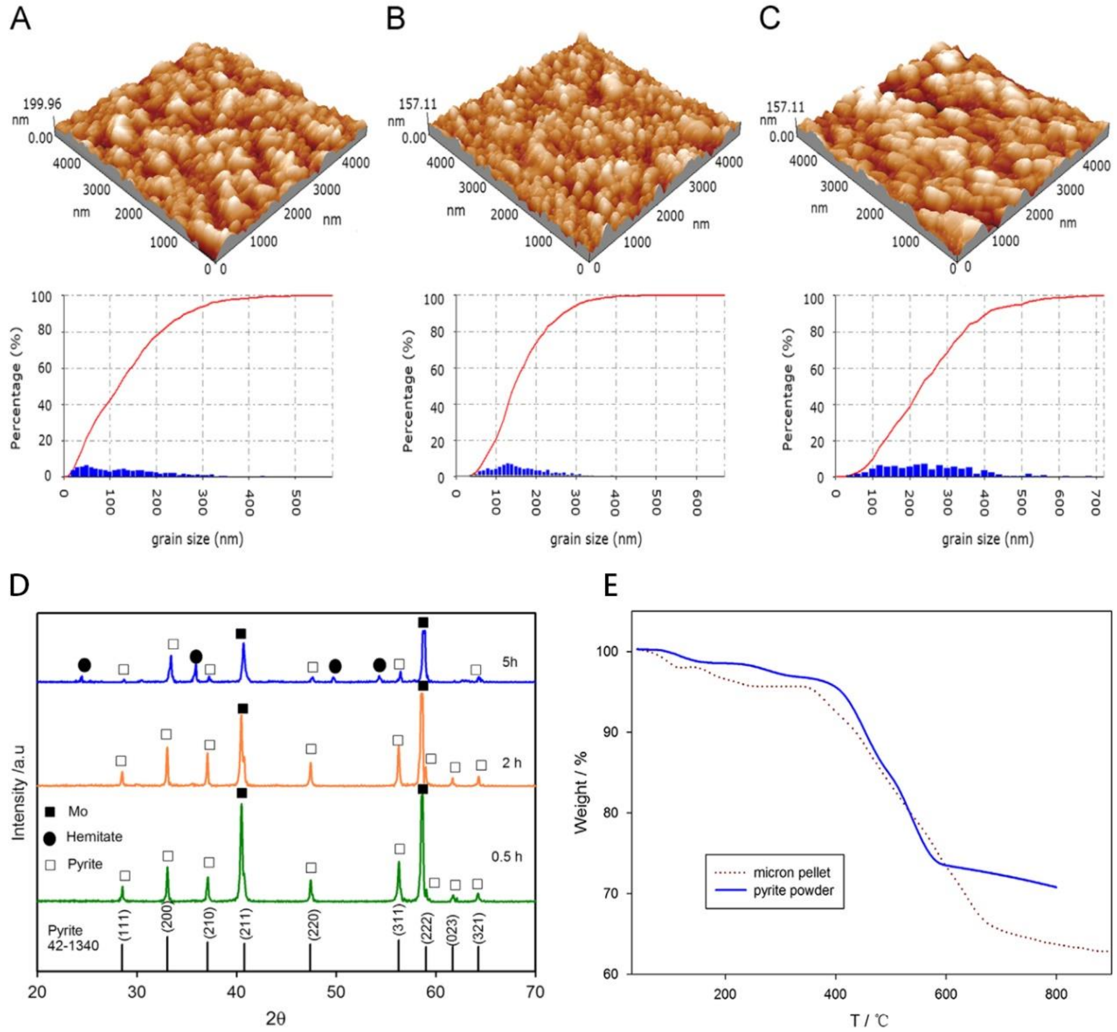

Figure S1. (a)(b)(c) Atomic Force microscope (AFM) morphologies and grain distributions of pyrite films after isothermal annealing for 0.5 h, 2h, and 5h. **(d)** X-ray diffraction (XRD) profiles of films annealed for 0.5 h, 2 h and 5 h at 773 K. **(e)** Thermal gravimetric analysis (TGA) of pyrite powder from pyrite films and the data of micron pyrite pellet was recorded in ref (1).

Wang et al. synthesized pyrite films with good crystallization, high activity area, high purity and good thermal stability by sulfuring electrodeposited iron sulfide films at 773 K under dynamic sulfur atmosphere and this prepared pyrite thin films with several superior physical properties could be used as an attractive cathode material for thermal batteries(2)**. The thermal stability of pyrite powder was slightly better than that of micron pyrite pellets.**

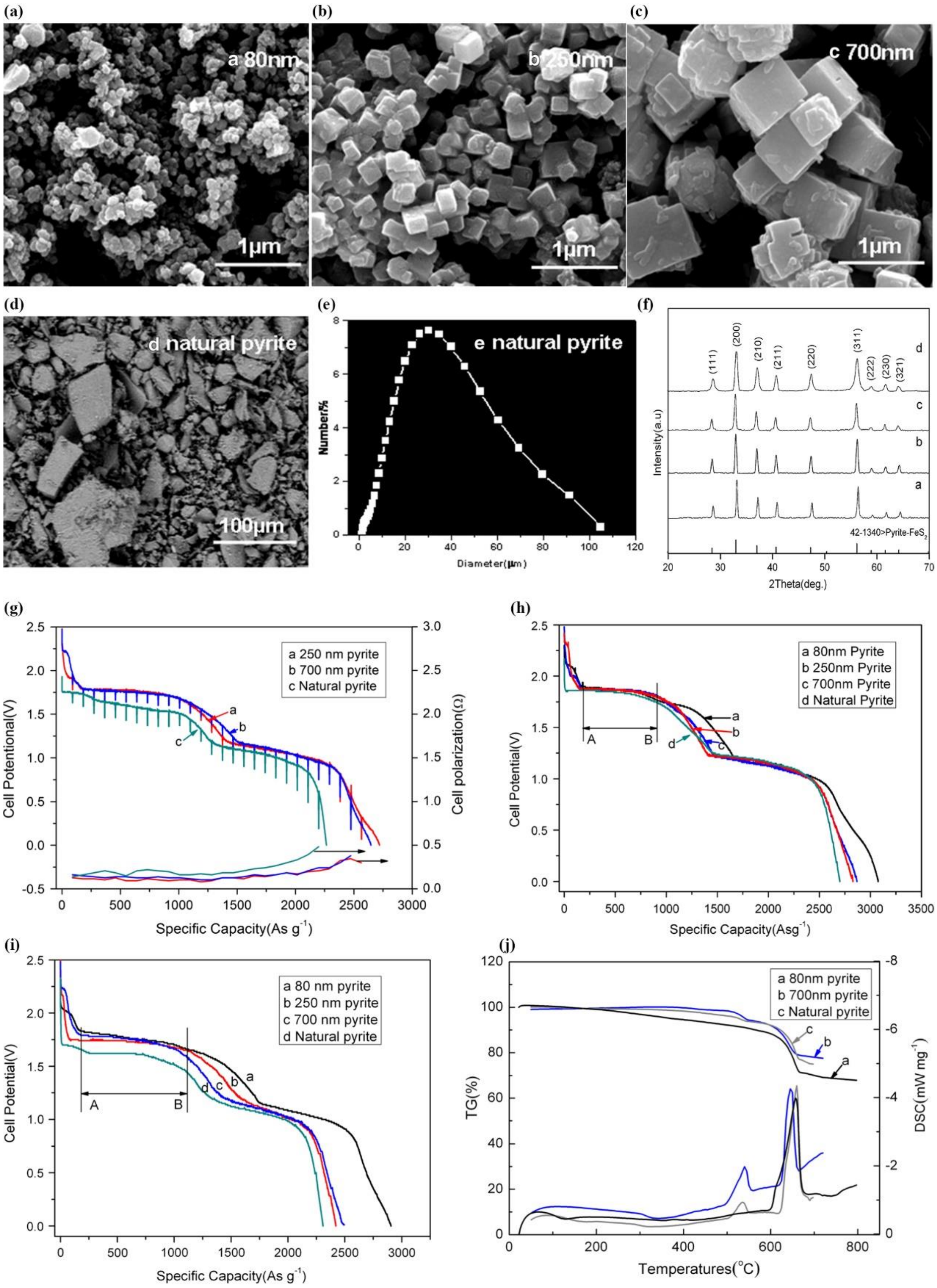

Figure S2. Scanning electron microscope (SEM) images of pyrite with different sizes **(a)** 80 nm; **(b)** 250 nm; **(c)** 700 nm; **(d)** natural counterpart; **(e)** Laser particle size analysis of natural counterpart. **(f)** X-ray diffraction (XRD) profiles of FeS_2: a 80 nm; b 250 nm; c 700 nm; d natural counterpart. **(g)** Polarization of the single-cell with synthetic and natural pyrite cathodes. Current density: 0.5 A/cm^2; pulse current density: 1 A/cm^2. **(h)** Discharge curves of natural and synthetic pyrite cathodes

with different particle sizes under 500 °C at 0.2 A/cm^2. **(i)** Discharge performance of natural and synthetic pyrite cathodes under 500 °C at 0.5 A/cm^2. **(j)** Thermogravimetry-differential scanning calorimetry (TG-DSC) analysis of natural and synthetic pyrite cathodes in a pure Ar atmosphere.

Yang et al. synthesized micro/nano-sized pyrite as cathode material by hydrothermal method and found that the electrochemical properties of the synthesized pyrite were improved compared with natural pyrite powder(3)**.**

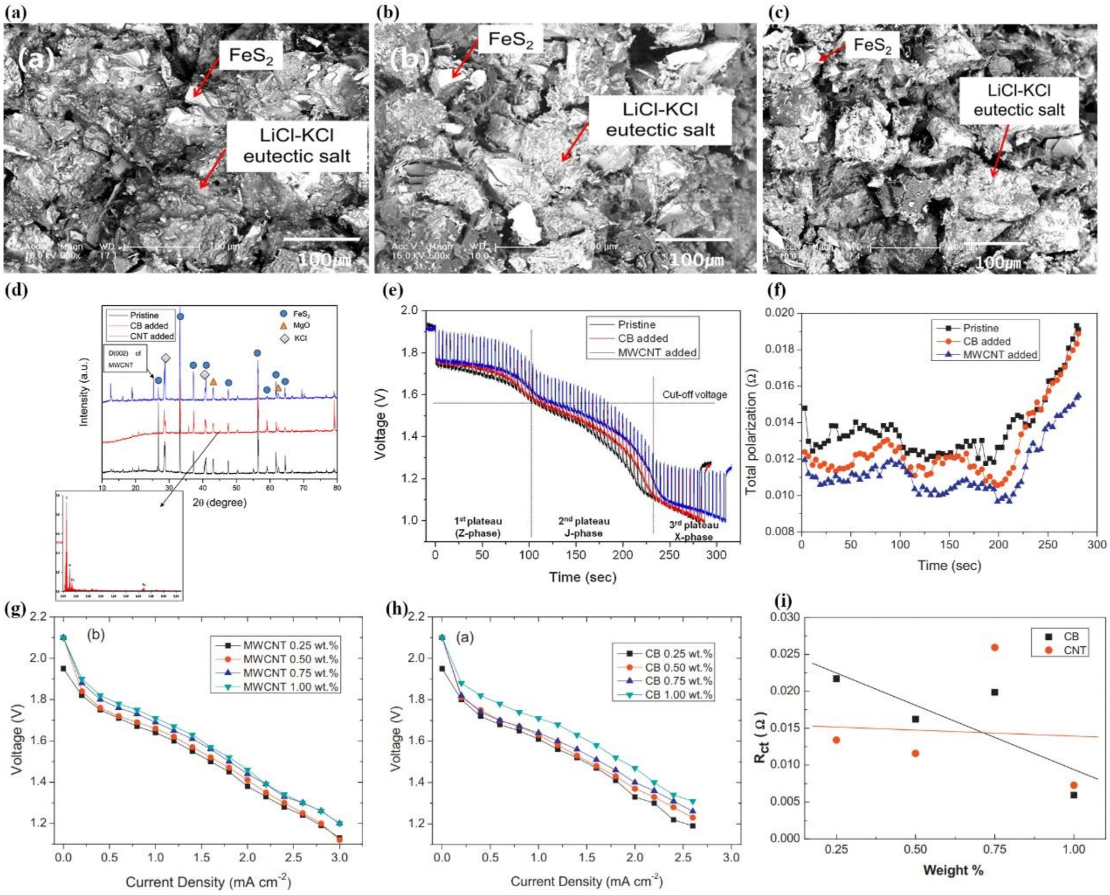

Figure S3. SEM images of **(a)** pristine, **(b)** 0.1 wt.% carbon black (CB)-added and **(c)** 0.1 wt.% multi-walled carbon nanotube (MWCNT)-added cathode materials. **(d)** XRD pattern of the pristine, 0.1 wt.% CB-added and 0.1 wt.% MWCNT-added cathode materials, and energy dispersive X-ray spectra of pyrite cathode material with CB addition. **(e)** Discharge performance of pristine, 0.1 wt.% CB-added and 0.1 wt.% MWCNT-added pyrite samples under single-cell test condition. **(f)** Total polarization results of the pristine, 0.1 wt.% CB-added and 0.1 wt.% MWCNT-added single thermal battery. Current density (in mA/cm^2) varies with Voltage (in V) for **(g)** CB-added and **(h)** MWCNT-

added pyrite samples from 0.25 to 1.0 wt.%. **(i)** Charge transfer resistance of thermal batteries with CB and MWCNT addition.

Choi reported that the addition of the conductive carbonaceous materials to pyrite electrode could improve the electrochemical performance of thermal batteries, which benefitted from the reduced charge transfer resistance by forming a conductive network between the pyrite particles(4)**.**

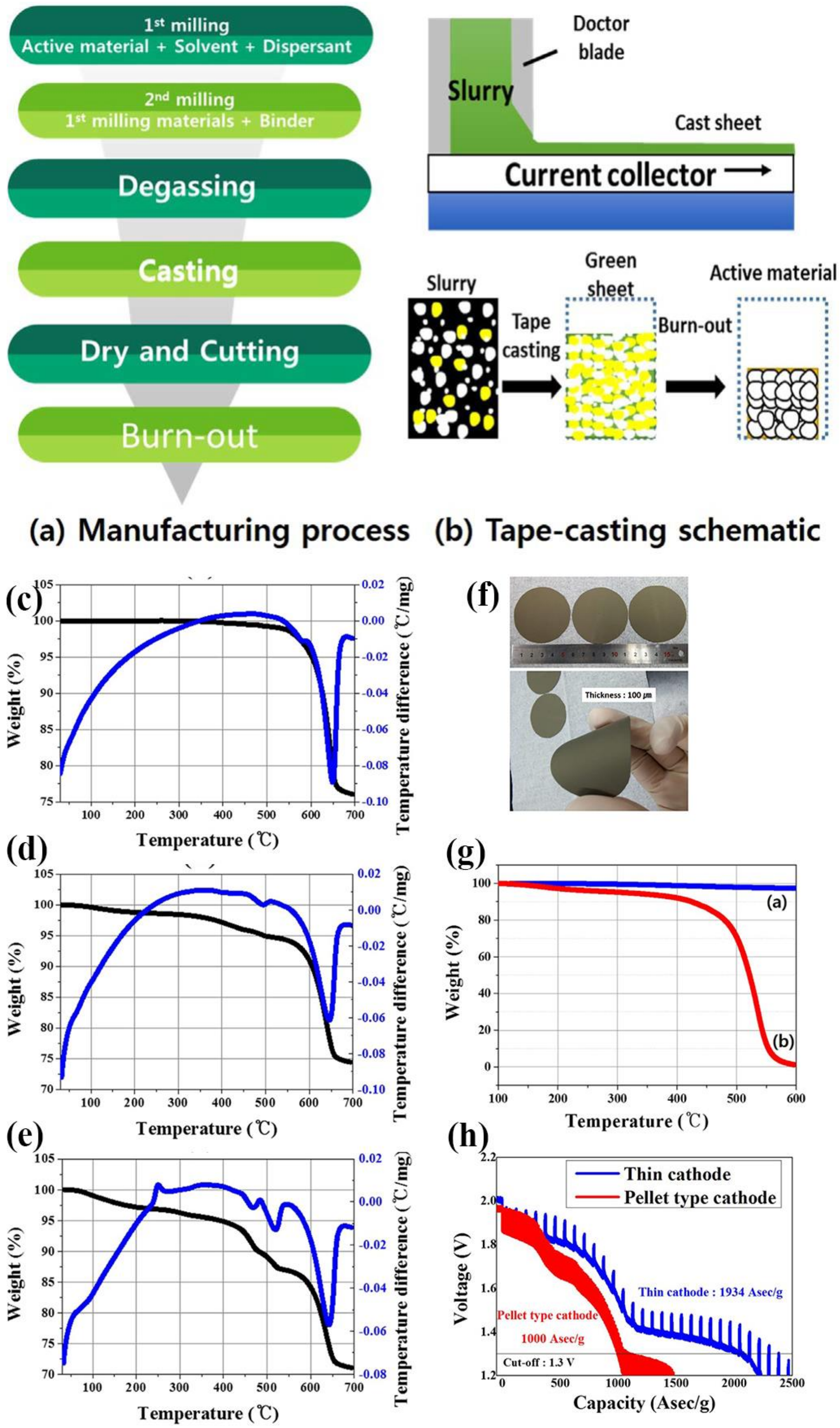

Figure S4. (a) The preparation process and **(b)** Schematic of preparing thin cathode by tape-casting. TGA-DTA results of **(c)** pristine FeS_2 powder and **(d)** the prepared FeS_2 powder by ball milled

method for 24 h. **(e)** 48 h-ball milled FeS_2 powder in solvent. **(f)** Photograph of the prepared thin cathode via a tape-casting process. TGA traces of binder materials **(g)** Silicic acid and **(h)** Polysiloxane.

Ko prepared thin cathodes for thermal batteries with good homogeneity and a reproducible thickness via a tape-casting process(5)**.**

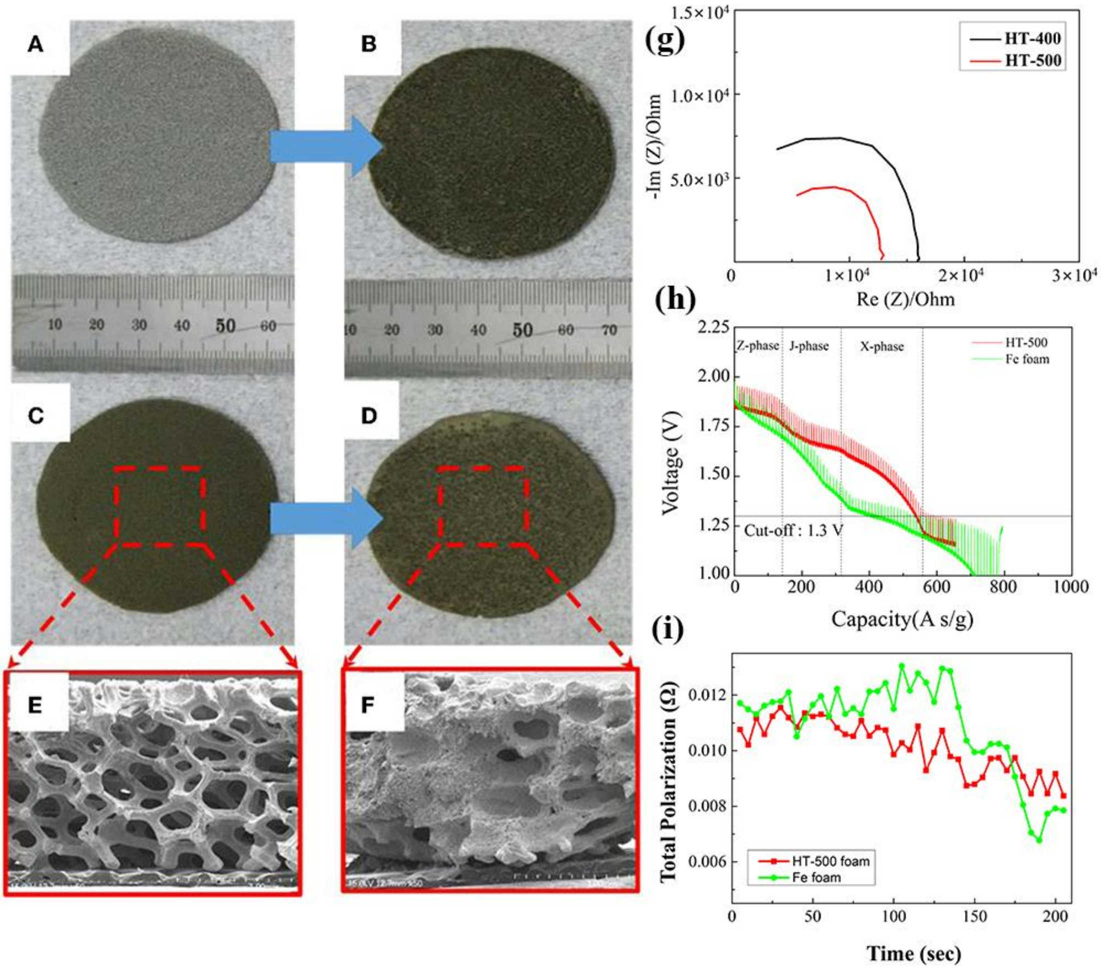

Figure S5. (a) Photograph of pure Fe foam before and **(b)** after impregnation with FeS_2 slurry. HT-500 foam **(c)** before and **(d)** after impregnation with FeS_2 slurry. Cross-section of the SEM image of HT-500 **(e)** before and **(f)** after impregnation with FeS_2 slurry. **(g)** Nyquist plots of HT-400 and HT-500 foams. **(h)** Discharge curves of cathodes using Fe and HT-500 foams. **(i)** Total polarization results of cathode using Fe and HT-500 foams.

Kim fabricated HT-500 foam via a sulfidation process for use as a thermal cathode frame(6)**. The discharge capacity of a single cell with the HT-500 foam**

was 538.38 A s/g, which was 1.3 times higher than that of the Fe foamed single cell.

2.2 CoS_2

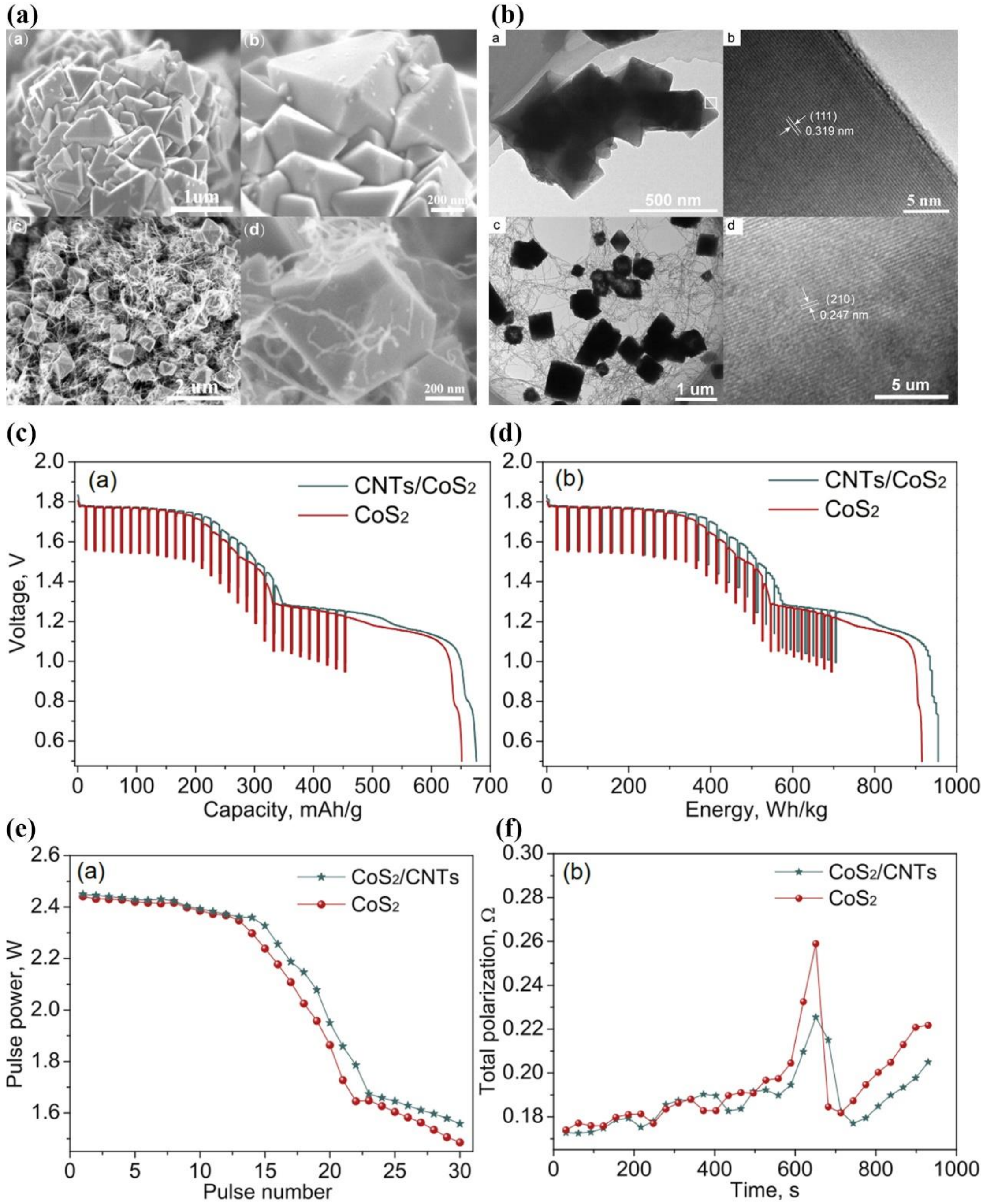

Figure S6. (a) SEM morphologies of CoS_2 (upper panel) and CoS_2/CNTs (bottom panel). **(b)** TEM morphologies of CoS_2 (upper panel) and CoS_2/CNTs (bottom panel). **(c)** Specific discharge capacity **(d)** energy, data of CoS_2 and CoS_2/CNTs at a background current of 100 mA/cm^2 and a pulse current

of 500 mA/cm^2 for 1 s every 30 s and for 30 pulses. **(e)** Pulse power and **(f)** polarization, data of CoS_2 and CoS_2/CNT.

By using hydrothermal growth method, Xie et al. prepared CoS_2 in the presence of carbon nanotubes (CNTs) as the cathode material of thermal battery(7)**. The octahedral crystalline particles emerged and were in agreement with the presence of cubic CoS_2. The pulse discharge performance of CoS_2/CNTs was superior than that of CoS_2.**

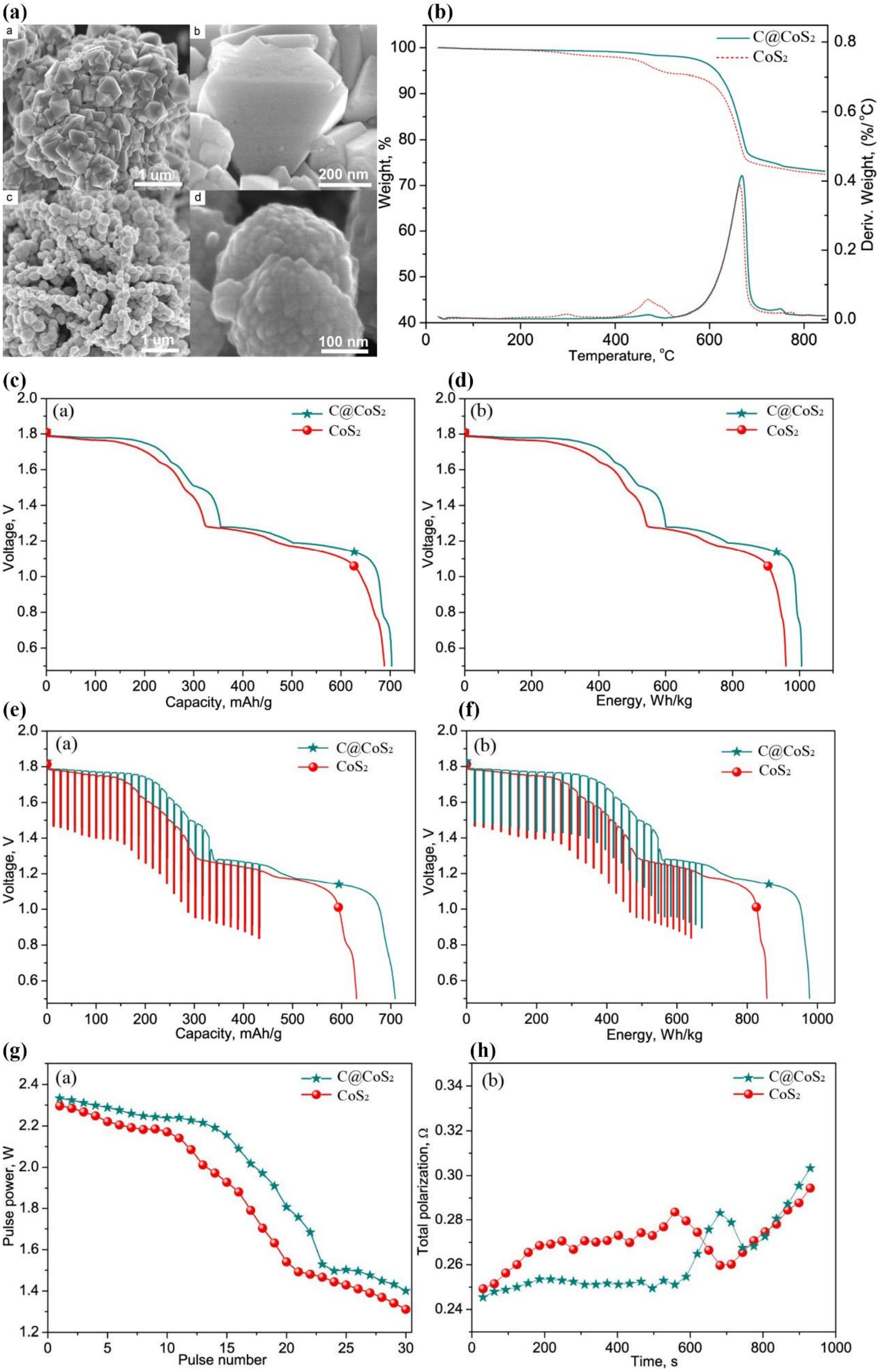

Figure S7. (a) SEM morphologies of bare CoS_2 (upper panel) and C@CoS_2 composite (bottom panel). **(b)** TGA of CoS_2 and C@CoS_2 kept in dry air for three months. **(c)** Discharge capacity and

(d) energy density of CoS_2 and $C@CoS_2$ when the current density is 100 mA/cm^2. **(e)** Pulse discharge capacity and **(f)** energy density of CoS_2 and $C@CoS_2$ composite when the current density is 100 mA/cm^2. **(g)** Pulse power and (h) polarization data of CoS_2 and $C@CoS_2$.

Xie et al prepared a novel carbon coated CoS_2 ($C@CoS_2$) cathode material via a facile one-pot hydrothermal method(8)**. The bare CoS_2 was octahedral crystal particle and the average particle size distribution was about 500 nm. The $C@CoS_2$ was composed of interconnected spherical particles with an average diameter of about 300 nm. The decomposition temperature of $C@CoS_2$ was 610 °C**(9)**, 60 °C higher than that of pyrite, 100 °C higher than that of synthesized FeS_2**(3) **and 200 °C higher than that of CoS_2 nanocrystal**(10)**. In-situ adsorption and carbonization of glucose contribute to homogeneous growth of CoS_2, resulting in lower particle size, which provided a larger specific surface area and facilitated contact between electrode material and electrolyte.**

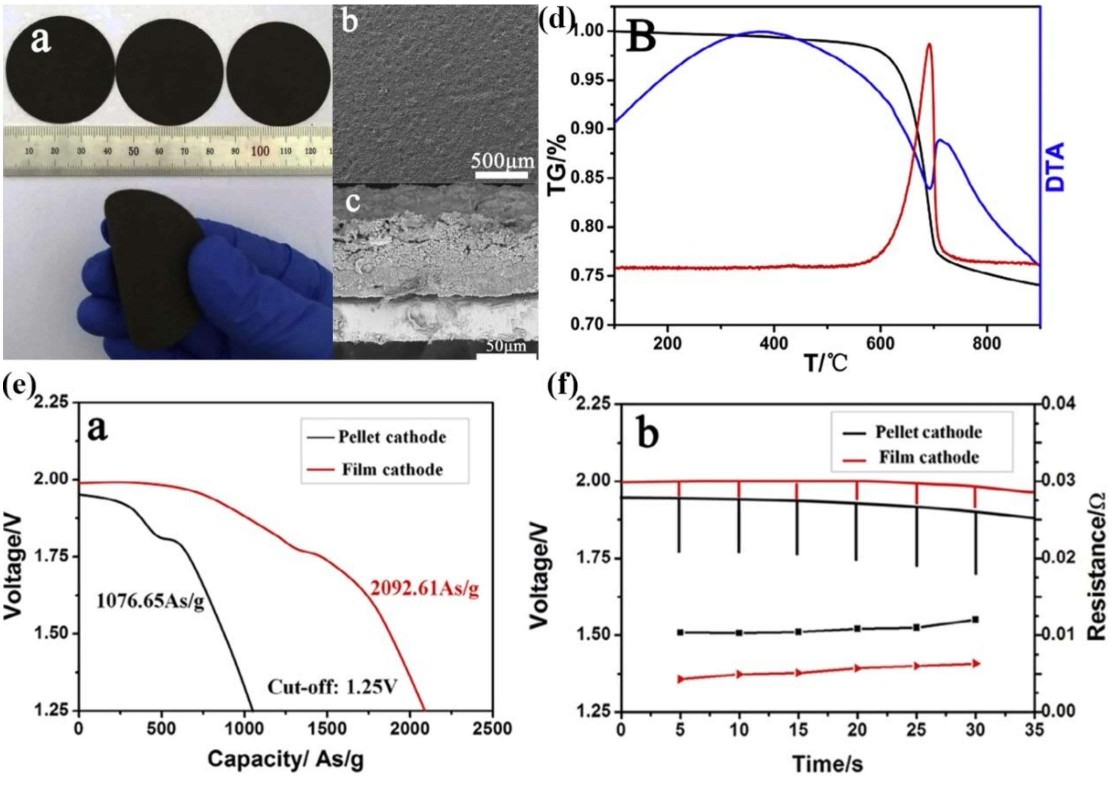

Figure S8. (a) Photograph and **(b)** SEM image of the surface. **(c)** cross-sectional morphologies of film cathodes synthesized via the screen-printing process. **(d)** TGA of the synthesized CoS_2 powders.

(e) Discharge curves of the film cathode and pellet cathode at 300 mA cm^{-2}. **(f)** Resistance changes curves of the film cathode and pellet cathode based single cell.

Hu prepared CoS_2 film cathode via the screening-printing method(11). **The thickness of the film cathode was 50 μm, which was thinner than that of pellet counterpart, suggesting that the film cathode gains good mechanical strength with good flexibility. The specific capacity of film cathode was 2092.61 As g^{-1}, which was 1015.96 As g^{-1} higher than that of pellet cathode.**

2.3 NiS_2

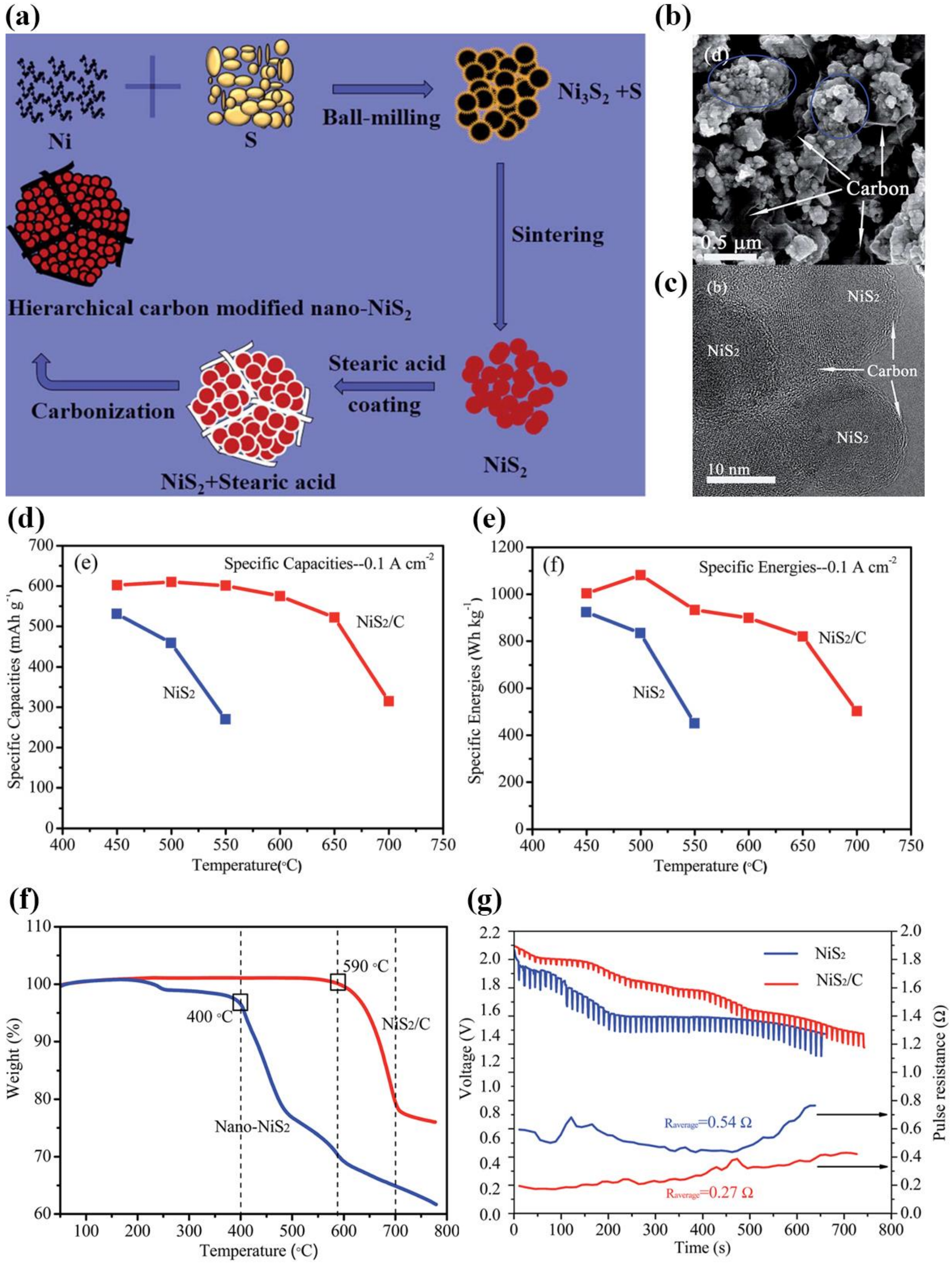

Figure S9. **(a)** Schematic of the preparation of NiS_2/C. **(b)** SEM image of NiS_2/C. **(c)** HRTEM of NiS_2. **(d)** Specific capacities and **(e)** specific energies of NiS_2 and NiS_2/C at 0.1 A/cm^2 at different discharge temperatures, until a minimum operating voltage of 1.4 V. **(f)** TGA of nano-NiS_2 and NiS_2/C. **(g)** Pulse discharges and resistances of the Li/NiS_2 cell before and after carbon coating at a

background current of 0.1 A/cm^2 and pulse current of 0.2 A/cm^2 for 1 s every 10 s.

Jin employed hierarchical carbon modification to the enhance the thermal stability and conductivity of nanostructured NiS_2(12). After carbon modification, the clusters can enhance electron and ion transport among the nanocrystals. Most of the clusters were connected by carbon mesh to further improve electron and ion transmission of nano-NiS_2.

2.4 Transition metal chloride

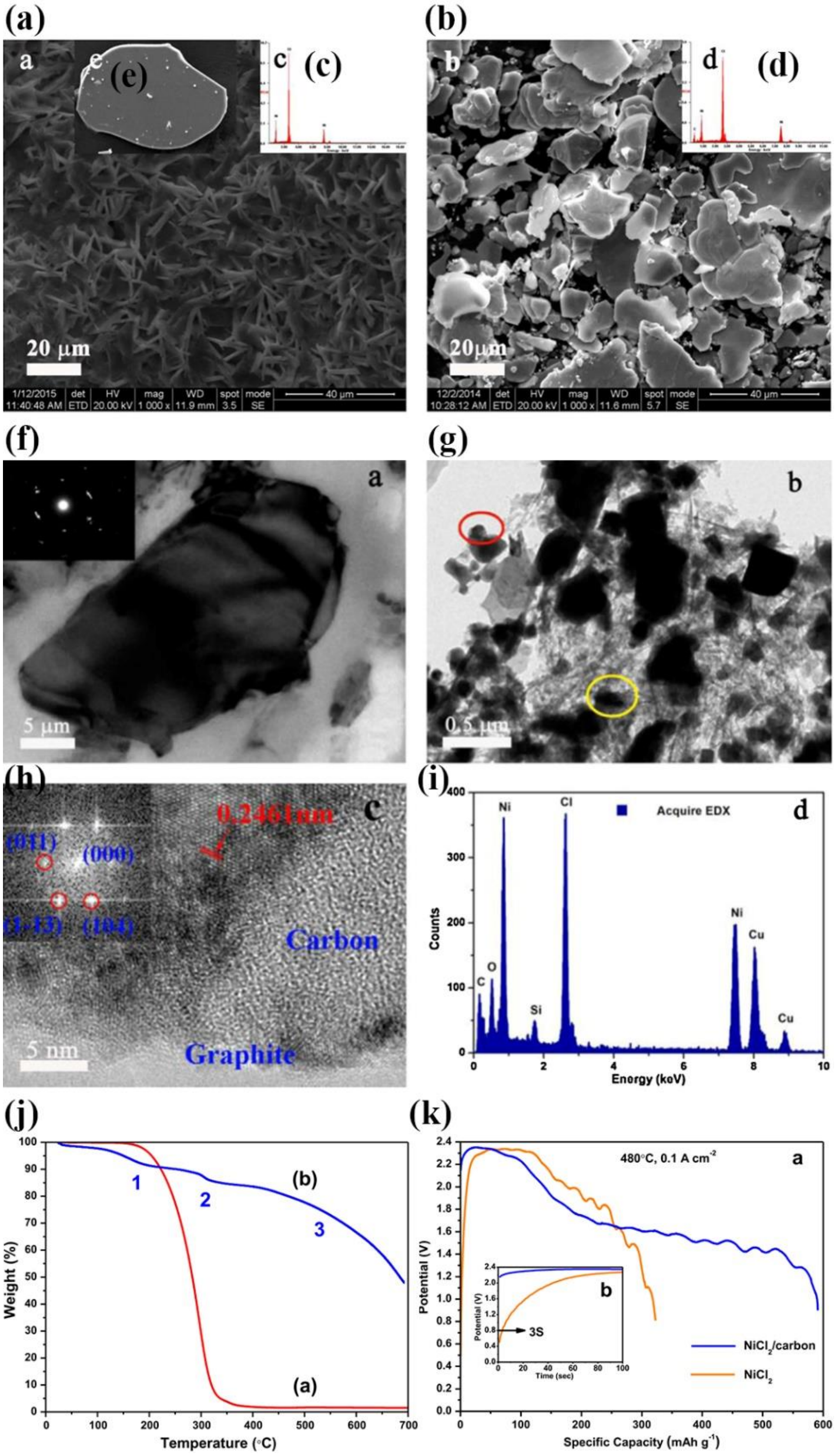

Figure S10. SEM images of **(a)**$NiCl_2$; **(b)** carbon coated $NiCl_2$; EDS analysis (inset) of **(c)** $NiCl_2$, **(d)** carbon coated $NiCl_2$; **(e)** sublimated $NiCl_2$ slice. TEM images and composition of $NiCl_2$ and carbon coated $NiCl_2$: **(f)** $NiCl_2$ and SAED, **(g)** carbon coated $NiCl_2$; **(h)** $NiCl_2$ high resolution image from red circle region in **(g)**; **(i)** EDS pattern from yellow circle region in **(g)**.

Jin prepared pure $NiCl_2$ by a vacuum sintering after sublimation and synthesized the carbon coated $NiCl_2$ via a simple solid-state reaction(13)**. The improvement of electrochemical performance of thermal battery after carbon coating.**

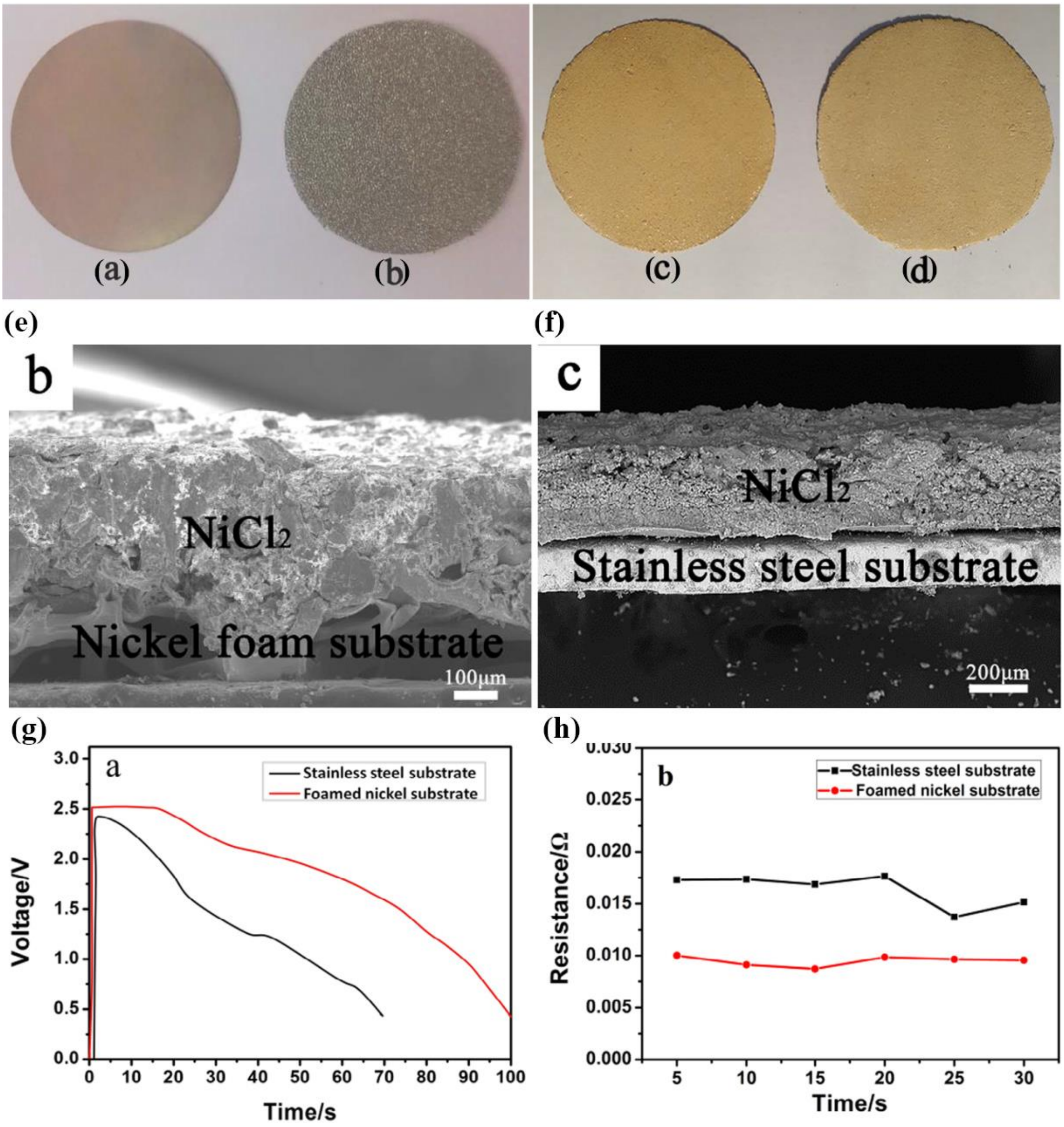

Figure S11. Photographs of $NiCl_2$ cathodes with stainless steel substrate **(a)** back, **(c)** front and Ni foam substrate **(b)** back, **(d)** front. SEM of the cross-sectional morphologies of **(e)** $NiCl_2$ with Ni foam and **(f)** $NiCl_2$ cathode with stainless steel substrate. **(g)** Discharge performance of thermal batteries with stainless steel substrate and Ni foam substrate at 300 mA cm^{-2}. **(h)** Resistance curves of $NiCl_2$ cell batteries with stainless steel substrate and Ni foam substrate.

Hu filled sublimated $NiCl_2$ into Ni foam instead of compact stainless steel(14). **$NiCl_2$ was fully filled into the porous Ni foam substrate, which can effectively enhance the electrical conductivity and the thermal conductivity of the cathode.**

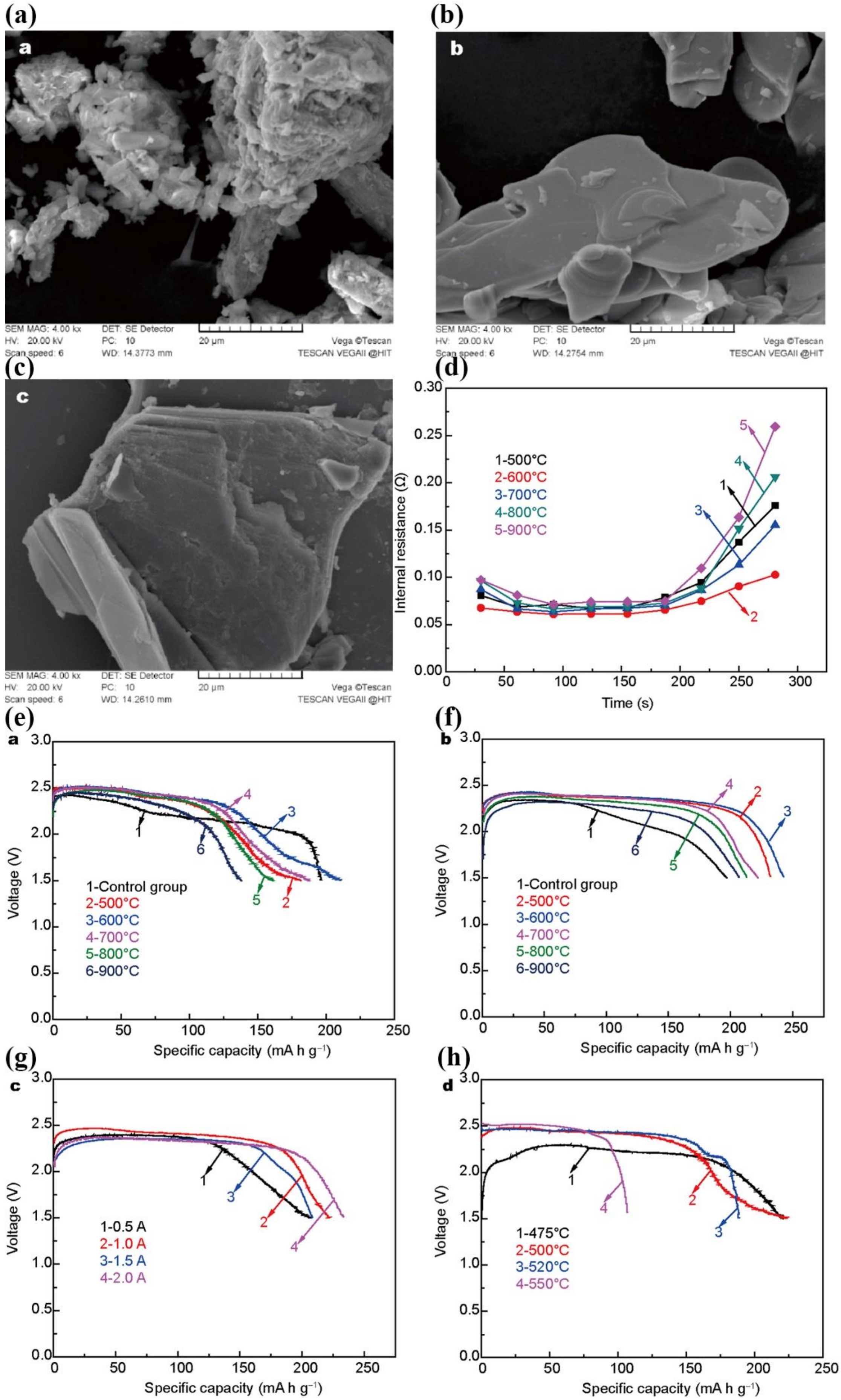

(a)
a
SEM MAG: 4.00 kx
HV: 20.00 kV
Scan speed: 6
DET: SE Detector
PC: 10
WD: 14.3773 mm
20 μm
Vega ©Tescan
TESCAN VEGAII @HIT
(b)
b
SEM MAG: 4.00 kx
HV: 20.00 kV
Scan speed: 6
DET: SE Detector
PC: 10
WD: 14.2754 mm
20 μm
Vega ©Tescan
TESCAN VEGAII @HIT
(c)
c
SEM MAG: 4.00 kx
HV: 20.00 kV
Scan speed: 6
DET: SE Detector
PC: 10
WD: 14.2610 mm
20 μm
Vega ©Tescan
TESCAN VEGAII @HIT
(d)
Internal resistance (Ω)
Time (s)
1-500°C
2-600°C
3-700°C
4-800°C
5-900°C
(e)
a
Voltage (V)
Specific capacity (mA h g⁻¹)
1-Control group
2-500°C
3-600°C
4-700°C
5-800°C
6-900°C
(f)
b
Voltage (V)
Specific capacity (mA h g⁻¹)
1-Control group
2-500°C
3-600°C
4-700°C
5-800°C
6-900°C
(g)
c
Voltage (V)
Specific capacity (mA h g⁻¹)
1-0.5 A
2-1.0 A
3-1.5 A
4-2.0 A
(h)
d
Voltage (V)
Specific capacity (mA h g⁻¹)
1-475°C
2-500°C
3-520°C
4-550°C

Figure S12. SEM morphologies of materials pre-dehydrated at **(a)** 270 °C, sintered at **(b)** 600 °C, sintered at **(c)** 900 °C. (scale bar: 20 μm) **(d)** Total internal resistance values of Li-B/LiCl-LiBr-LiF/$NiCl_2$ cells at 500 °C and 2.0-4.0 Å with different high-temperature-sintered materials. Discharge performance of Li-B/LiCl-LiBr-LiF/$NiCl_2$ cells at 500 °C and constant currents of **(e)** 0.5 A and **(f)** 2.0 A with different high-temperature-sintered materials, and different constant currents **(g)**, and different discharge temperatures **(h)** with 600 °C-sintered materials.

Liu proposed a novel two-step variable temperature solid-state method to remove the crystal water from $NiCl_2$ hexahydrate(15)**. The aggregation of grains with small size into larger particles was benefited to increase the tap density of the cathode material due to better interfacial contact of the material and better particle-particle contact among the $NiCl_2$ particles.**

2.5 Transition metal fluorides

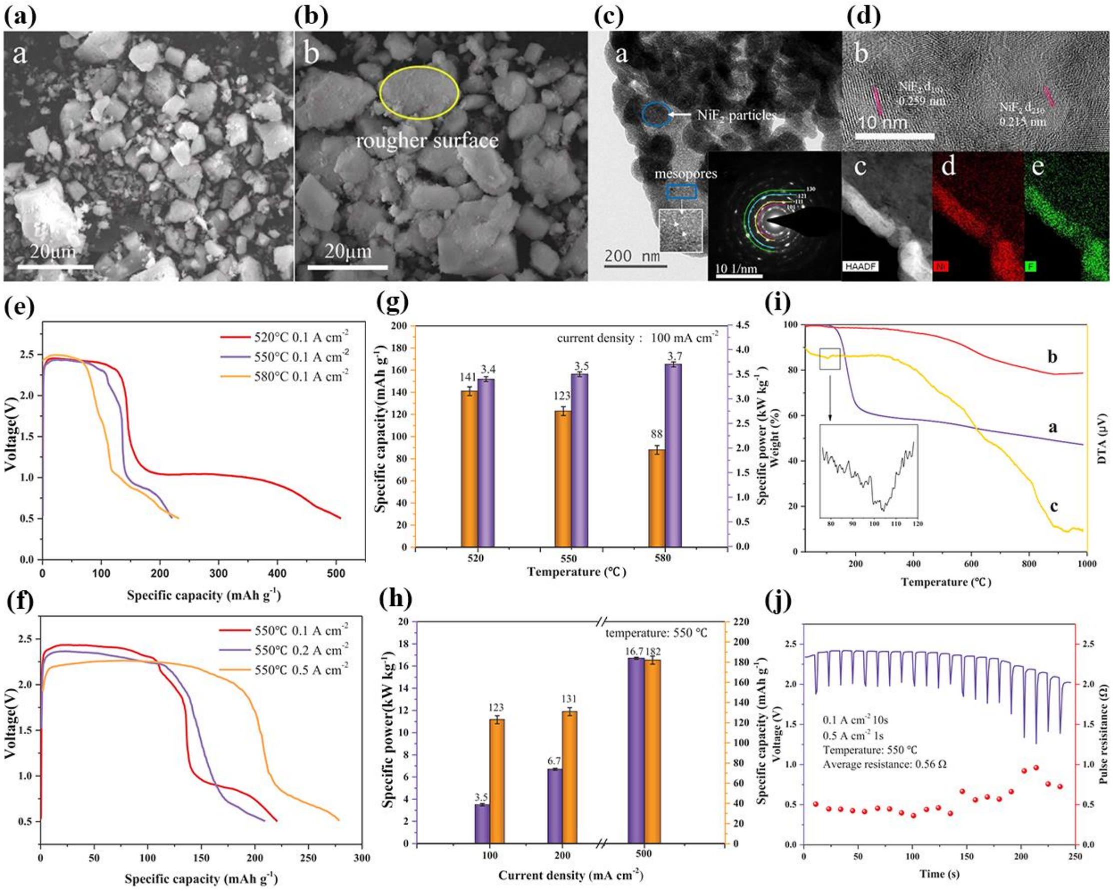

Figure S13. SEM morphologies of **(a)** $NiF_2 \cdot 4H_2O$ and **(b)** NiF_2. TEM morphologies of **(c)** NiF_2. The inserted image is electron diffraction pattern of NiF_2. **(d)** HRTEM image of NiF_2. The inserted images are STEM-Highangle Annular Dark Field (HAADF) image and STEM-EDS elemental mapping of Ni and F. The discharge performance of single NiF_2 cathode-based thermal batteries at **(e)** 0.1 A cm^{-2} under different temperatures and at **(f)** different current densities under the same temperature of 550°C. **(g, h)** the corresponding specific capacity and specific power of NiF_2. **(i)** TGA of $NiF_2 \cdot 4H_2O$, NiF_2 and corresponding DTA curve of NiF_2 sample from ambient to 1000 °C under Ar. **(j)** Pulse discharge and the resistance of the NiF_2/Li-B cell at a background current of 0.1 A cm^{-2} and pulse current of 0.5 A cm^{-2} for 1 s every 10 s.

Chang prepared pure NiF_2 via a direct and simple two-step dehydration method from commercia $NiF_2 \cdot 4H_2O$(16). **NiF_2 particles tended to agglomerate during sintering, which induced a porous structure with a high specific surface.**

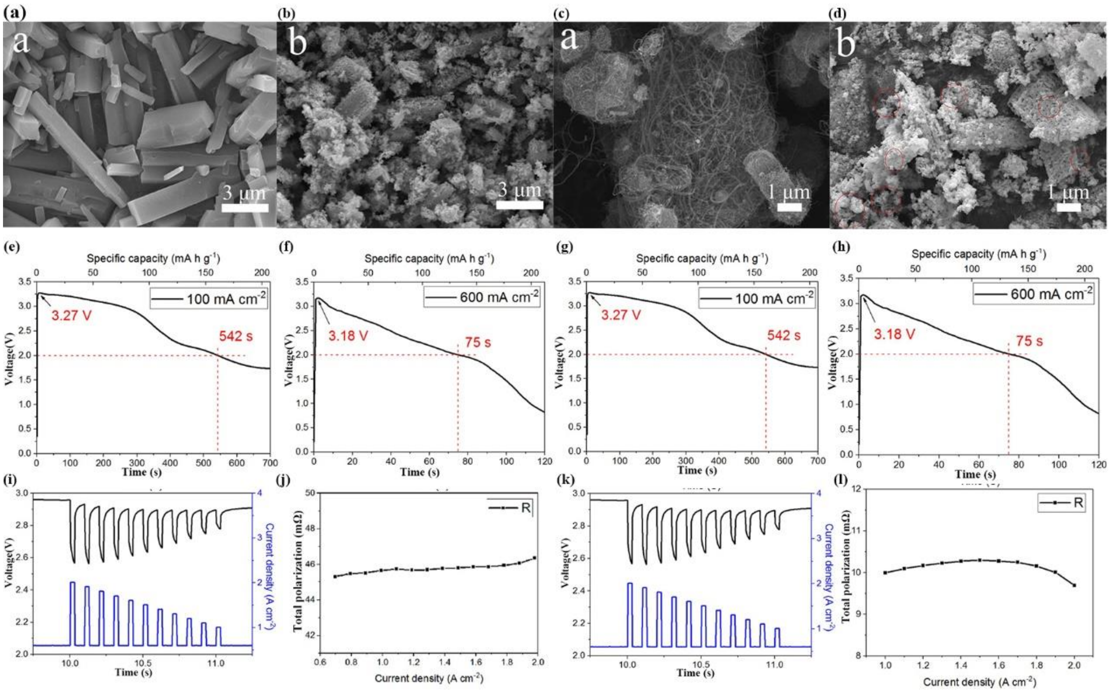

Figure S14. SEM morphologies of **(a)** $FeF_3 \cdot 3H_2O$ precursor; **(b)** FeF_3, **(c)** MWCNTs and **(d)** the mixed FeF_3 sample at 10000x. **(e)** Discharge curve of Li-B/FeF_3 single cell at 100 mA cm^{-2} and **(f)** 600 mA cm^{-2}. **(g)** Discharge curve of Li-B/FeF_3-MWCNT single cell at 100 mA $cm^{-2}$and **(h)** 600 mA cm^{-2}. Pulse current and corresponded changes of discharge voltage of **(i)** Li-B/FeF_3 single cell and **(k)** Li-B/FeF_3-MWCNT single cell. Total polarization of **(j)** Li-B/FeF_3 single cell and **(l)** Li-B/FeF_3-MWCNT single cell.

Guo synthesized the anhydrous FeF_3 by a liquid-phase method combined with a thermal treatment process(17)**. The large bandgap of pristine FeF_3 can hinder the transfer electrons, inducing more self-discharge reaction than electrode reaction. The conductive MWCNTs can facilitate the electrons transfer, and the electrode reaction prioritized the self-discharge reaction.**

Table S1 Summary of the performance of cathode materials for thermal batteries.

Cathode	Anode	Electrolyte	Cathode type/Thickness (μm)	Phase	The preparation method of cathode	Specific capacity (mAh/g)	Discharge Temperature (°C)	Cut-off (V)	OCV (V)	Voltage plateau (V)	Thermal stability (°C)	Ref.
FeS_2	Li-Si	LiCl-KCl	Nano-particle/0.70	Cubic	Hydrothermal	364.44	500	0.88	2.50	1.75/1.13	623	(3)
FeS_2	Li-Si	LiCl-KCl	Thin film/100	-	Tape-casting	537.24	500	1.30	2.0	1.75/1.4	580	(5)
FeS_2	Commercial	-	Foam/1600	Cubic	Sulfidation	149.55	500	1.30	1.87	1.82/1.68/1.48	-	(6)
FeS_2	Li-Si	LiCl-KCl	Particle/98	Cubic	Mixing	-	500	1.00	1.90	1.70/1.45/1.05	-	(4)
FeS_2	Li-Si	LiCl-KCl	Thin film/50	-	Tape-casting	640.28	500	1.30	2.00	1.85/1.40	580	(18)
CoS_2	Li-Si	LiCl-KCl	Nanocrystal/-	Cubic	Hydrothermal	200.70	500	1.70	1.80	1.75/1.50/1.20	640	(7)
CoS_2	Li-Si	LiCl-KCl	Pellet/-	Cubic	Hydrothermal	235.80	500	1.70	1.84	1.78/1.30	650	(8)
CoS_2	Li-Si	LiCl-LiBr-LiF	Thin film/50	Cubic	Screen printing	581.28	500	1.25	1.99	1.90/1.76	650	(11)
CoS_2	Li-B	LiF-LiCl-LiBr	Particle/19.70	-	Solid-state reaction	258.00	520	1.40	1.80	1.75/1.65	724.30	(19)
$Fe_{0.5}Co_{0.5}S_2$	Li-Si	LiCl-LiBr-LiF	Thin film/50	Cubic	Liquid phase	520.25	-	1.25	2.04	2	620	(20)
NiS_2	Li-Si	KCl-LiCl	Nanocrystal/-	Cubic	-	335.00	520	1.20	1.84	1.71/1.44/1.24	-	(21)
NiS_2	Li-B	LiCl-LiBr-LiF	Nanocrystal/0.085	Cubic	Mechano-chemical activation/sintering	794.00	500	0.50	2.20	1.98/1.80/1.40	400	(22)
NiS_2	Li-B	LiCl-LiBr-LiF	Nanocrystal/0.04	Cubic	Solid-state reaction	610.00	500	1.40	2.00	2.00/1.60	590	(12)
MoS_2	Li-Si	LiF-LiCl-LiBr	Nanosheet/0.01	Mixed 2H/1T	Hydrothermal	281.25	450	1.00	1.20	1.10	700	(23)
WS_2	Li-B	LiF-LiCl-LiBr	Nanosheet/0.01-0.03	2H	Solid-state reaction	334.70	500	1.00	1.43	1.39	1200	(24)
ZrS_3	Li-Si	LiCl-KCl	Pellet/-	Cubic/spinel	Solid-state reaction	357.00	500	1.00	1.80	1.70	700	(25)
$NiCl_2$	Li-B	LiF-LiCl-LiBr	Nanocrystal/0.32	-	Rheological phase method	591.00	480	0.80	2.35	2.30/1.50	-	(13)
$NiCl_2$	Li-Si	LiCl-LiBr-LiF	Foam/400	-	Screen printing	180.17	-	1.50	2.55	-	-	(14)
$NiCl_2$	Li-B	LiCl-LiBr-LiF	Particle/-	-	Two-step variable-temperature	242.84	600	1.50	2.24	2.42	-	(15)
$NiCl_2$	Li-B	LiCl-LiBr-LiF	Particle/187.50	-	Hydrogen reduction	188.00	550	25.00	32.5	31.25	500	(26)
$PbCl_2$	Li	LiCl-KCl	-	-	Simple tablet method	168.00	450	0.50	1.95	1.88	-	(27)
NiF_2	Li-B	LiF-LiBr-LiCl	Particle/20.00	Tetragonal	Two-step dehydration	181.50	550	2.00	1.95	2.25	900	(16)
FeF_3	Li-B	LiCl-LiF-LiBr	Particle/5~10	-	Liquid-phase method	160.70	500	2.00	3.27	3.13/2.13	800	(17)
CuV_2O_6	Li-Al	LiCl-KCl	Pellet/1000	Triclinic	Solid-state method	220.00	525	2.00	3.50	-	600	(28)

References:

1. Au M. Nanostructured thermal batteries with high power density. *J Power Sources* (2003) 115(2):360-6. doi: 10.1016/S0378-7753(02)00627-4.
2. Wang X, Wang G, Chen J, Zhu X, Tian J, Jiang C, et al. Pyrite thin films prepared for thermal batteries via sulfuring electrodeposited iron sulfide films: Structure and physical properties. *Mater Lett* (2013) 110:144-7. doi: 10.1016/j.matlet.2013.07.107.
3. Yang Z, Liu X, Feng X, Cui Y, Yang X. Hydrothermal synthesized micro/nano-sized pyrite used as cathode material to improve the electrochemical performance of thermal battery. *J Appl Electrochem* (2014) 44(10):1075-80. doi: 10.1007/s10800-014-0724-9.
4. Choi Y, Cho S, Lee Y. Effect of the addition of carbon black and carbon nanotube to FeS2 cathode on the electrochemical performance of thermal battery. *J Ind Eng Chem* (2014) 20(5):3584-9. doi: 10.1016/j.jiec.2013.12.052.
5. Ko J, Kim IY, Jung HM, Cheong H, Yoon YS. Thin cathode for thermal batteries using a tape-casting process. *Ceram Int* (2017) 43(7):5789-93. doi: 10.1016/j.ceramint.2017.01.126.
6. Kim IY, Woo SP, Ko J, Kang S, Yoon YS, Cheong H, et al. Binder-Free Cathode for Thermal Batteries Fabricated Using FeS2 Treated Metal Foam. *Front Chem* (2020) 7. doi: 10.3389/fchem.2019.00904.
7. Xie S, Deng Y, Mei J, Yang Z, Lau W, Liu H. Facile synthesis of CoS2/CNTs composite and its exploitation in thermal battery fabrication. *Composites Part B: Engineering* (2016) 93:203-9. doi: 10.1016/j.compositesb.2016.03.038.
8. Xie S, Deng Y, Mei J, Yang Z, Lau W, Liu H. Carbon coated CoS2 thermal battery electrode material with enhanced discharge performances and air stability. *Electrochim Acta* (2017) 231:287-93. doi: 10.1016/j.electacta.2017.02.068.
9. Masset PJ, Guidotti RA. Thermal activated (“thermal”) battery technology. *J Power Sources* (2008) 177(2):595-609. doi: 10.1016/j.jpowsour.2007.11.017.
10. Ji Y, Liu X, Liu W, Wang Y, Zhang H, Yang M, et al. A facile template-free approach for the solid-phase synthesis of CoS2 nanocrystals and their enhanced storage energy in supercapacitors. *Rsc Adv* (2014) 4(91):50220-5. doi: 10.1039/C4RA08614G.
11. Hu J, Chu Y, Tian Q, Wang J, Li Y, Wu Q, et al. Film cathode for thermal batteries using a screen-printing process. *Mater Lett* (2018) 215:296-9. doi: 10.1016/j.matlet.2017.12.114.
12. Jin C, Fu L, Zhu J, Yang W, Li D, Zhou L. A hierarchical carbon modified nano-NiS2 cathode with high thermal stability for a high energy thermal battery. *J Mater Chem a* (2018) 6(16):7123-32. doi: 10.1039/C8TA00346G.
13. Jin C, Zhou L, Fu L, Zhu J, Li D. Synthesis and discharge performances of NiCl2 by surface modification of carbon coating as cathode material of thermal battery. *Appl Surf Sci* (2017) 402:308-13. doi: 10.1016/j.apsusc.2017.01.034.
14. Hu J, Chu Y, Tian Q, Guo S, Yang M, Wang X, et al. Electrochemical properties of the NiCl2 cathode with nickel foam substrate for thermal batteries. *Mater Lett* (2017) 207:198-201. doi: 10.1016/j.matlet.2017.07.082.
15. Liu W, Liu H, Bi S, Cao L, Sun Y. Variable-temperature preparation and performance of NiCl2 as a cathode material for thermal batteries. *Science China Materials* (2017) 60(3):251-7. doi: 10.1007/s40843-016-9003-x.
16. Chang Q, Luo Z, Fu L, Zhu J, Yang W, Li D, et al. A new cathode material of NiF2 for thermal

batteries with high specific power. *Electrochim Acta* (2020) 361:137051. doi: 10.1016/j.electacta.2020.137051.

17. Guo SN, Guo H, Wang X, Zhu Y, Hu J, Yang M, et al. Iron Trifluoride as a High Voltage Cathode Material for Thermal Batteries. *J Electrochem Soc* (2019) 166(15):A3599-605. doi: 10.1149/2.0371915jes.
18. Jaehwan Ko IYKH. Organic binder-free cathode using FeS2-MWCNTs composite for thermal batteries. *J Am Ceram Soc* (2017) 100(10):4435-41. doi: 10.1111/jace.14991.
19. Xie Y, Liu Z, Ning H, Huang H, Chen L. Suppressing self-discharge of Li–B/CoS2 thermal batteries by using a carbon-coated CoS2 cathode. *Rsc Adv* (2018) 8(13):7173-8. doi: 10.1039/C7RA13071F.
20. Hu J, Zhao L, Chu Y, Tian Q, Wang J, Li Y, et al. Preparation and electrochemical properties of a new Fe0.5Co0.5S2 cathode material for thermal batteries. *J Alloy Compd* (2018) 762:109-14. doi: 10.1016/j.jallcom.2018.05.118.
21. Payne JL, Percival JD, Giagloglou K, Crouch CJ, Carins GM, Smith RI, et al. In‐Situ Thermal Battery Discharge using NiS2 as a Cathode Material. *Chemelectrochem* (2017) 4(8):1916-23. doi: 10.1002/celc.201700095.
22. Jin C, Zhou L, Fu L, Zhu J, Li D, Yang W. The acceleration intermediate phase (NiS and Ni 3 S 2) evolution by nanocrystallization in Li/NiS 2 thermal batteries with high specific capacity. *J Power Sources* (2017) 352:83-9. doi: 10.1016/j.jpowsour.2017.03.119.
23. Zheng X, Zhu Y, Sun Y, Jiao Q. Hydrothermal synthesis of MoS2 with different morphology and its performance in thermal battery. *J Power Sources* (2018) 395:318-27. doi: 10.1016/j.jpowsour.2018.05.092.
24. Guo S, Guo H, Wang X, Zhu Y, Yang M, Zhang Q, et al. Synthesis and electrochemical performance of WS2 nanosheet for thermal batteries. *Mater Lett* (2019) 249:81-3. doi: 10.1016/j.matlet.2019.04.030.
25. Giagloglou K, Payne JL, Crouch C, Gover RKB, Connor PA, Irvine JTS. Zirconium Trisulfide as a Promising Cathode Material for Li Primary Thermal Batteries. *J Electrochem Soc* (2016) 163(14):A3126-30. doi: 10.1149/2.1351614jes.
26. Tian Q, Wang J, Xiang W, Zhao J, Guo H, Hu J, et al. Fabrication of the Ni-NiCl2 Composite Cathode Material for Fast-Response Thermal Batteries. *Front Chem* (2021) 9:679231. doi: 10.3389/fchem.2021.679231.
27. Chen F, Jiang C, Xu L, Li X, Shen Q. High utilization rate thermal batteries using PbCl2 as a cathode material. *Mater Lett* (2021) 299:130018. doi: 10.1016/j.matlet.2021.130018.
28. Hillel T, Ein-Eli Y. Copper vanadate as promising high voltage cathodes for Li thermal batteries. *J Power Sources* (2013) 229:112-6. doi: 10.1016/j.jpowsour.2012.11.128.
